# Locus coeruleus degeneration is associated with orthostatic hypotension in Parkinson’s disease and multiple system atrophy

**DOI:** 10.1007/s10286-025-01164-2

**Published:** 2025-10-31

**Authors:** Paul A. Beach, Sierra Hyland, Xiangchuan Chen, Daniel E. Huddleston

**Affiliations:** 1 Department of Neurology, Emory University School of Medicine, Woodruff Memorial Building, 6th Floor, 101 Woodruff Circle, Atlanta, GA 30322 USA; 2 National Eye Institute, National Institutes of Health, Bethesda, MD USA

**Keywords:** Orthostatic hypotension, Parkinson’s disease, Multiple system atrophy, Locus coeruleus, Neuromelanin-MRI

## Abstract

**Purpose:**

We compared locus coeruleus (LC) structural integrity, as measured by neuromelanin-sensitive magnetic resonance imaging (NM-MRI), between patients with Parkinson’s disease (PD) and those with multiple system atrophy (MSA) and tested whether orthostatic hypotension (OH) impacted differences in LC volume between PD and MSA. Substantia nigra (SNc) volumes were compared between these groups to determine whether effects observed in LC were specific to that structure. Last, we tested whether LC integrity is associated with orthostatic hemodynamic responses.

**Methods:**

Presence or absence of (±)OH was determined with active stand testing. Automated segmentation of LC and SNc volumes took place using NM-MRI. Structural volumes were first compared between PD and MSA groups and, second, after stratification by OH status. Last, correlations between LC volumes and orthostatic vitals were calculated.

**Results:**

Of 71 patients with PD, 19 were (+)OH. Of 19 patients with MSA, 12 were (+)OH. LC volumes were larger in PD than MSA (*p* = 0.002), and LC volumes in PD(−)OH were larger than PD(+)OH and MSA(±)OH (*p* < 0.05). All comparisons involving SNc were nonsignificant. Primarily in PD(−)OH, LC volumes correlated negatively with supine mean arterial pressure (MAP) and positively with supine heart rate. In PD(+OH) and MSA(+)OH, lower LC volumes were correlated with greater orthostatic falls in MAP.

**Conclusions:**

Similar levels of LC neurodegeneration were observed in PD(+)OH and MSA(±)OH. Therefore, LC measurement may be useful to differentiate PD(−)OH from MSA. Lower LC volumes additionally correlated with greater drops in MAP in both PD(+)OH and MSA(+)OH, suggesting that LC neurodegeneration may contribute to OH in both conditions.

**Supplementary Information:**

The online version contains supplementary material available at 10.1007/s10286-025-01164-2.

## Introduction

Parkinson’s disease (PD) and multiple system atrophy (MSA) are neurodegenerative conditions that cause parkinsonism and autonomic failure [1, 2]. A hallmark manifestation of autonomic failure is orthostatic hypotension (OH), which occurs at some point in up to ~ 80% of patients with MSA [3] and 30–50% of patients with PD [4, 5]. For these reasons, differential diagnosis between PD and parkinsonian MSA is often challenging. This is especially the case in scenarios of PD with early autonomic failure, which occurs in upward of 5–10% of cases [6, 7], and early parkinsonian MSA *without* autonomic failure, which occurs in upward of 38% of patients [3]. These initial overlapping features likely contribute to misdiagnosis rates of ~ 20–40% [8, 9]. The recently updated diagnostic criteria for MSA concluded that clinically established MSA, indicating the highest level of clinical specificity [2, 10], requires at least one magnetic resonance imaging (MRI) marker in addition to core clinical features. However, classic MRI findings associated with MSA are often not present early in disease [11], are non-specific [12], or are not readily identifiable from a qualitative standpoint [13, 14]. Quantifiable neuroimaging biomarkers would be highly beneficial toward increasing the sensitivity of this tier of clinical diagnostic certainty.

Synucleinopathies cause depletion of central catecholamines through neurodegeneration of their striatal, midbrain, and pontine sources, including the substantia nigra pars compacta (SNc) and locus coeruleus (LC) [15]. Neuromelanin MRI (NM-MRI) is capable of visualizing the magnetic resonance (MR) signal of these neuromelanin-rich regions. With this modality, neurodegeneration, or loss of catecholaminergic function, in the SNc or LC is inferred on the basis of loss of contrast-to-signal ratio intensity or reduction in structural volume [16]. This inference is supported by pathological studies of the SNc and LC that show a direct association between loss of NM-containing neurons and postmortem NM-MRI signal intensity [17–19]. Prior NM-MRI studies have found that, in comparison to healthy individuals, patients with PD and and those with MSA demonstrate decreased NM-MRI signal in the SNc [20, 21] and LC [21–24]. However, MSA is a more centrally focused and rapidly progressive neurodegenerative disease, compared with PD, and is thought to cause particularly greater brainstem noradrenergic neurodegeneration than PD [25, 26]. NM-MRI-based quantitative approaches, particularly of the LC, may thus be a promising tool to help differentiate PD from MSA.

Comparisons of SNc between PD and MSA consistently show similar degrees of NM-MRI signal between PD and MSA [23, 27, 28]. However, amongst the few studies that have compared LC measures using NM-MRI between PD and MSA, there are conflicting findings of lesser [23], greater [27, 29], or equal [30] NM-MRI signal in MSA, compared with PD. We propose that a previously unaccounted for factor, the presence of autonomic failure, as exemplified by OH, may explain these conflicting findings. While OH in PD is considered to result primarily from postganglionic sympathetic neurodegeneration [31, 32], those with early OH may have a more aggressive neurodegenerative phenotype [33, 34] as demonstrated by greater central noradrenergic neurochemical and structural abnormalities [15], including in the LC [35, 36], compared with PD without OH. However, it is unclear whether OH in PD is linked to a nonspecific increase in central neurodegeneration or a more specific impact on noradrenergic structure and function.

We therefore sought to determine, first, whether volume-based NM-MRI measures of the LC can differentiate between clinically defined PD and MSA and whether the presence of OH in PD impacts this differentiation. However, because the LC is implicated in autonomic function and prior work has demonstrated a correlation between LC structure and function with orthostatic hemodynamic responses in PD [35, 36], we hypothesized that PD with OH would be associated with a greater degree of LC neurodegeneration and that LC volume would correlate with orthostatic blood pressures in PD and MSA. Third, we hypothesized that an increase in central catecholaminergic neurodegeneration found in PD with OH would be specific to LC. To test this last hypothesis, we also compared SNc volumes between groups, with and without OH stratification.

## Methods

### Subjects and clinical evaluation

This prospective study was approved by the Emory Institutional Review Board. Data were collected between 2016 and 2023. All participants provided written informed consent. In this study, 71 participants with PD and 19 with MSA were recruited from the Emory University Movement Disorders Clinic. All participants with PD and MSA were diagnosed by a fellowship-trained movement disorders neurologist according to the UK Parkinson’s Disease Society Brain Bank and Second Consensus Criteria for MSA diagnosis [37, 38]. Study exclusion criteria were: (1) drug-induced parkinsonism; (2) a history of multiple sclerosis, territorial ischemic stroke, hemorrhagic stroke, epilepsy, parenchymal brain tumor, moderate-to-severe head trauma, hydrocephalus, other neurodegenerative diseases; or (3) treatment with dopamine-blocking drugs. Vasoactive medications were not explicitly held during study procedures.

Demographic information including age, sex, education, race, and disease duration was collected for each participant. Dopaminergic medication strength and daily doses were recorded to calculate levodopa-equivalent daily doses (LEDDs) using the Parkinson’s measurement toolbox (https://www.parkinsonsmeasurement.org/toolBox/levodopaEquivalentDose.htm). A subset of participants had additional health history and nonparkinsonian medications recorded. Disease severity, including nonmotor and motor impacts on daily life and motor examination, was evaluated using the Movement Disorders Society Unified Parkinson’s Disease Rating Scale (MDS-UPDRS) parts I–III [39]. Cognition was assessed using the Montreal Cognitive Assessment (MoCA) [40]. The Scales for Outcomes in Parkinson’s Disease—Autonomic (SCOPA-AUT) evaluated autonomic symptoms experienced within the prior month [41]. The Rapid Eye Movement (REM) Sleep Behavior Disorder Questionnaire (RBDSQ) evaluated symptoms of REM sleep behavior disorder [42]. Patients were separated into RBDSQ groups on the basis of cutoff scores of ≥ 6, which were previously shown to have high sensitivity and specificity for polysomnogram-confirmed rapid eye movement sleep behavior disorder (RBD) in PD [43]. Both motor examination and orthostatic testing occurred after the participant took their typical dose of dopaminergic medications (medication “ON” state). As available, information on cardiovascular and other vasoactive medication use that could impact orthostatic hemodynamic responses was extracted. Orthostatic vitals (systolic blood pressure [SBP], diastolic blood pressure [DBP], mean arterial blood pressure [MAP], and heart rate [HR]) were evaluated using an active stand test. Supine blood pressure (BP) was measured after the participant rested in the supine position for several minutes. After standing, BP and HR were measured at 1, 3, and 5 min. Supine hypertension was determined on the basis of supine SBP ≥ 140 mmHg or DBP ≥ 90 mmHg [44]. Criteria for OH were based on standard clinical consensus criteria of a sustained fall in SBP ≥ 20 mmHg and/or DBP ≥ 10 mmHg within 3 min of standing [45]. We additionally included individuals with delayed OH, in which a pathologic fall in BP occurred only at minute 5 [46]. Given the possible influence of vasoactive medications taken prior to orthostatic testing, we further scrutinized orthostatic responses indicative of OH as being primarily neurogenic on the basis of two possible criteria: first, a ratio of HR/SBP change of 0.5 or less on average across the 5 min of standing measurement [47, 48] and/or having an average fall in SBP ≥ 30 mmHg across the 5 min of measurement. Inclusion within an OH subgroup required the presence of at least one of these criteria.

### MRI scanning and image analysis

All participants underwent MRI scanning with a Prisma-Fit 3 T scanner (Siemens Medical Solutions, Malvern, PA, USA) at Emory University using a 64-channel receive-only coil. NM-MRI data were acquired using an MT-prepared two-dimensional (2D) gradient echo (GRE) sequence under two protocols: protocol A—echo time (TE)/repetition time (TR) of 2.99/370 ms, 416 × 512 imaging matrix, voxel size of 0.39 × 0.39 × 3 mm^3^, 15 contiguous slices, 7 measurements, flip angle (FA) of 40°, 610 Hz/pixel receiver bandwidth, magnetization transfer contrast (MTC) pulses (300°, 1.2 kHz off resonance, and 10 ms duration), and scan time 17 min 12 s; and protocol B—TE/TR of 3.08/378 ms, 416 × 512 imaging matrix, voxel size: 0.39 × 0.39 × 3 mm^3^, 15 contiguous slices, 4 measurements, FA of 40°, 445 Hz/pixel receiver bandwidth, MTC pulses (300°, 1.5 kHz off resonance, and 10 ms duration), and scan time 10 min 30 s. A T1-weighted magnetization prepared MPRAGE sequence (TE/TR = 2.46/1900 ms, inversion time = 900 ms, FA = 9°, and voxel size = 0.8 × 0.8 × 0.8 mm^3^) was used for common space registration. On the sagittal T1-weighted images, the GRE scan slices were positioned perpendicular to the dorsal edge of the brain stem at midline along the fourth ventricle, starting from the lower pons (below the most caudal extent of the LC), covering the SNc and LC.

MRI data were processed using the FMRIB Software Library (FSL) [49] and Analysis of Functional NeuroImages (AFNI) [50]. NM-MRI images were motion-corrected by registering the separately acquired measurements to a single image using the FMRIB Linear Image Registration Tool (FLIRT) [51, 52] and then averaged. These images were then used for analysis. Next, individual subject space and common space transformations took place using an approach previously published by our group. [53]

SNc and LC volumes were segmented with an automated thresholding approach previously described by our group [54]. A reference region of interest (ROI) in each cerebral peduncle was generated in individual subject space and used to identify suprathreshold voxel intensities in SNc and LC. The mean and standard deviation (SD) of the signal intensities were determined for the reference ROIs. Voxels that were 2.57-SD or 2.88-SD greater in intensity than the mean intensity in the reference region were identified as SNc or LC, respectively. Thresholding was restricted to the anatomic location of SNc and LC using probabilistic standard space masks with a previously described approach. [55]

### Statistical analysis

Continuous variables related to demographics and clinical data underwent testing for normality using the Shapiro–Wilk test on the basis of diagnosis and by subgroup stratification (i.e., PD with and without OH and MSA with and without OH). Parametric data were compared, statistically, with independent-samples *t*-tests, while nonparametric data were compared with Mann–Whitney *U* testing. Chi-squared testing compared categorical data between groups, including relative proportions of each study group under the two NM-MRI protocols. LC and SNc volumes were initially compared with Mann–Whitney *U* tests. A significant main effect between groups (PD and MSA) was followed up with between-subgroup comparisons stratified by OH presence or absence using independent samples one-way analysis of variance (ANOVA; Kruskal–Wallis testing) with post hoc comparisons as appropriate. One participant with MSA was cerebellar in phenotype, and a sensitivity analysis was performed in comparing LC and SNc volumes between groups with this individual censored before repeating the analyses. In addition, influence of beta- and alpha-blocker medications reported by patients with OH was investigated with a sensitivity analysis whereby these individuals were censored before repeating the analysis. We separately evaluated the possible influence of scanner protocol and relevant clinical variables (age, disease duration, and RBDSQ group) on the main effect differences between PD and MSA with parametric (univariate analysis of covariance) or nonparametric (Quade’s nonparametric analysis of covariance) testing as appropriate. Correlational relationships between supine MAP and HR and average orthostatic changes in MAP and HR (across each measure during active stand testing) were examined with two-tailed Spearman correlation analyses. For all analyses, *p* < 0.05 was considered statistically significant. Unadjusted *p*-values are presented unless a family-wise error (FWE) or false discovery rate (FDR) correction is otherwise noted.

## Results

### Group and subgroup characteristics

A total of 90 individuals (*N* = 71 PD and *N* = 19 MSA) were analyzed for this study. Of these, there were 52 patients with PD without (−)OH, 19 with PD with (+)OH, 7 with MSA (−)OH, and 12 with MSA (+)OH. Among the patients with MSA, 18 had parkinsonian MSA and 1 had cerebellar MSA. Summary demographic and clinical characteristics of the sample, and statistical comparisons between primary (PD and MSA) and subgroups stratified by presence/absence of OH, are presented in Table 1. PD and MSA groups were well matched for age, sex, education level, and race (*p* > 0.05). Those with PD had a longer disease duration (*p* = 0.05) and took greater amounts of daily levodopa or related medications (*p* = 0.008). Several motor (MDS-UPDRS part II, *p* < 0.001, and part III, *p* = 0.003) and nonmotor symptom scale scores (MDS-UPDRS parts Ia, *p* = 0.04, and Ib, *p* < 0.001; SCOPA-AUT total and its cardiac domain, *p* < 0.001; and RBDSQ, *p* = 0.02) were higher in patients with MSA than PD, and cognitive function was reduced in MSA compared with PD (MoCA, *p* < 0.001). Supine MAP was higher in MSA (*p* = 0.01), as was the average fall in MAP after standing (*p* = 0.002). However, rates of supine hypertension (HTN) were similar between the primary groups, as was supine HR and its average orthostatic change. Detailed primary and subgroup orthostatic hemodynamic responses are presented in Supplementary Table S1.

**Table 1:**
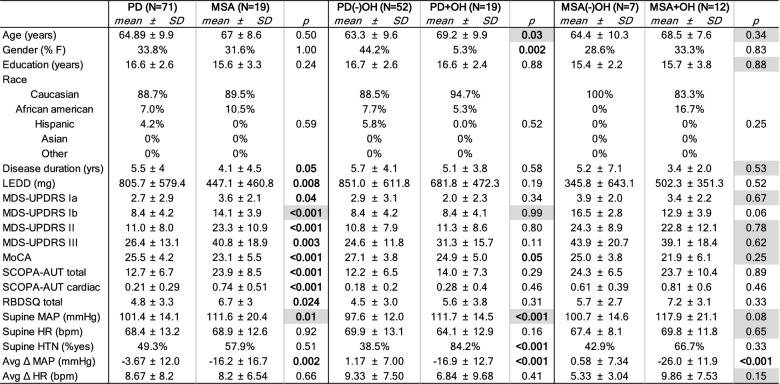
Clinical and demographic information

Supine HTN defined as SBP ≥ 140 mmHg and/or DBP ≥ 90 mmHg while supine, Δ reflects postural change subtracting stand–supine, and Avg reflect average change in orthostatic vital across minutes 1, 3, and 5. Bold: two-tailed *p* < 0.05 between groups (uncorrected). *p* values shaded gray reflect independent samples *t*-test results. Otherwise, *p* values reflect Mann–Whitney *U* results or chi-squared testing for categorical data

*PD* Parkinson disease, *MSA* multiple system atrophy, *OH* orthostatic hypotension, *%F* percentage female, *LEDD* levodopa equivalent doses, *MDS-UPDRS* Movement Disorder Society Unified Parkinson’s Disease Rating Scale, *MoCA* Montreal Cognitive Assessment, *SCOPA-AUT* Scales for Outcomes in Parkinson’s Disease—Autonomic, *RBDsq* = Rapid Eye Movement Sleep Behavior Disorder Symptoms Questionnaire, *min* minute, *SBP* systolic blood pressure, *DBP* diastolic blood pressure, *MAP* mean arterial pressure, *HR* heart rate, *HTN* hypertension

In comparing subgroups stratified by presence or absence of OH, the PD (+)OH group was older than PD (−)OH (*p* = 0.03), there were fewer numbers of females in the PD (+)OH group (*p* = 0.002), and MoCA scores were generally lower in the PD (+)OH group (*p* = 0.05). The PD (+)OH group also had a higher supine MAP (*p* < 0.001), greater rates of supine HTN (*p* = 0.002), and a greater average fall in MAP while standing (*p* < 0.001). Between the MSA subgroups, there was a greater average orthostatic fall in MAP in the MSA (+)OH group (*p* < 0.001), a tendency for MSA (−)OH to report greater intensities of overall nonmotor symptoms (MDS-UPDRS Ib, *p* = 0.06), and a tendency for there to be a higher supine MAP in MSA (+)OH (*p* = 0.08).

Roughly half of all participants had data available regarding medication use impacting cardiovascular function (other than dopaminergic medications) and health risk factors that could impact autonomic function. Relevant summary statistics are presented in Supplementary Table S2, but statistical comparisons were not computed given the frequency of missing data. Across PD and MSA, generally, similar relative proportions of participants reported antihypertensive use (35.1% and 28.6%, respectively) or having had a history of hypertension (37.8% and 42.9%, respectively). However, greater percentages of the PD (+)OH group reported taking antihypertensives than the MSA (+)OH group (63.4% and 33.3%, respectively). Among those with available data indicating use of antihypertensives, four patients in the PD (+)OH group were on beta-blockers or alpha-blockers. Very few patients with data available reported taking anti-OH medications, though one patient with PD who did not demonstrate OH during standing reported anti-OH medication use. Relatively few patients with PD (16.2%) and MSA (14.3%) reported a history of diabetes. Distributions of the two NM-MRI protocols by whole sample, by diagnosis, and by diagnosis stratified by OH presence can be found in Supplementary Table S3. Chi-squared testing found no difference in the proportions of groups by diagnosis (*p* = 0.90) or as stratified by the presence of OH (*p* = 0.67).

### LC and SNc comparisons between primary groups and subgroups

**Fig. 1:**
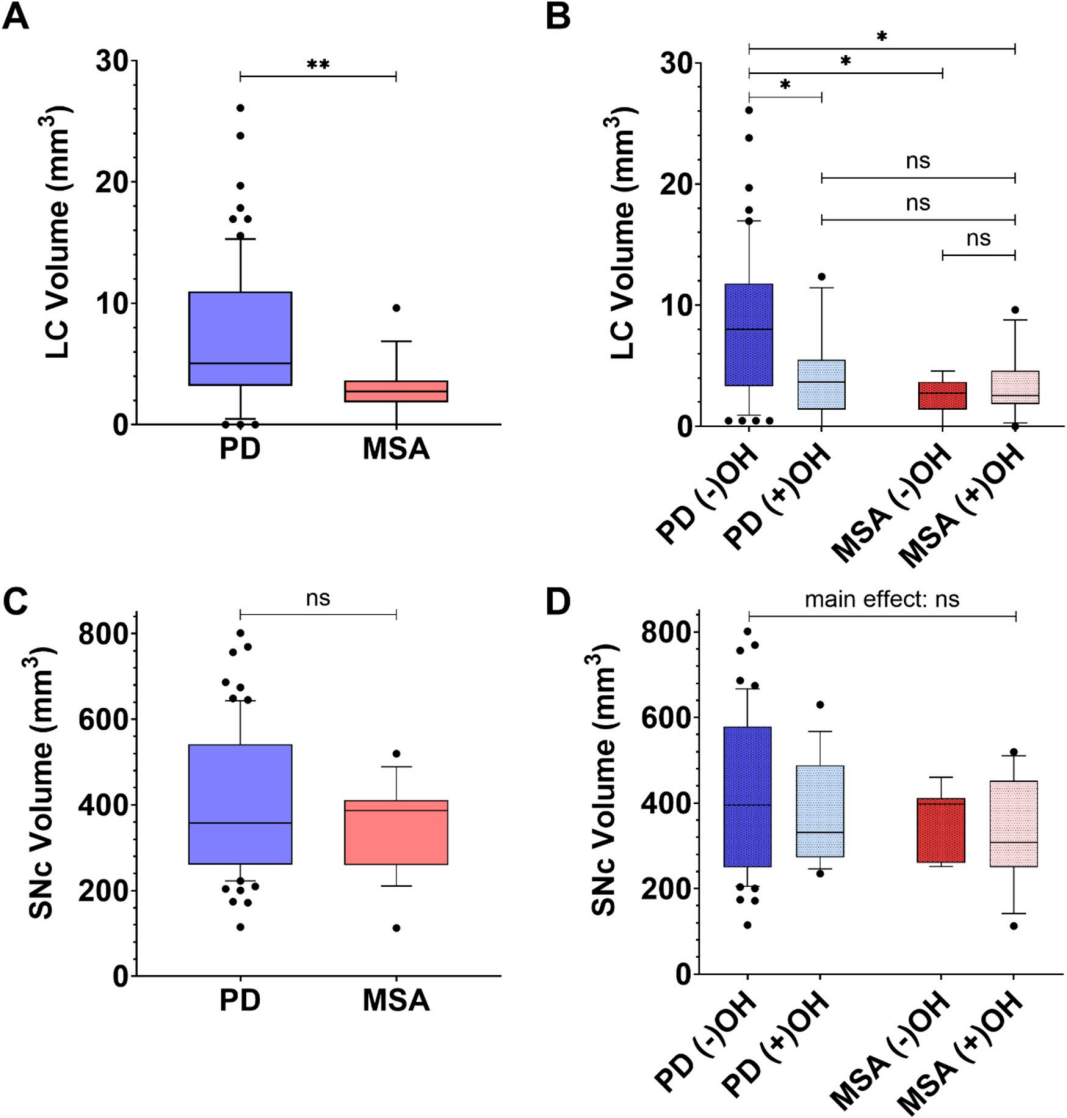
Box and whisker plot comparisons of locus coeruleus (LC) and substantia nigra pars compacta (SNc) volumes (mm^3^) by diagnosis (Parkinson’s disease [PD] and multiple system atrophy [MSA]—plots **A** and **C**), and across those without (−) and with (+) orthostatic hypotension (OH—plots **C** and **D**). **A** patients with MSA demonstrate smaller LC volumes than PD. **B** Stratified by OH presence, PD (−)OH demonstrates larger LC volumes than both MSA subgroups as well as PD (+)OH. **C** PD and MSA groups had similar SNc volumes, irrespective of presence of OH (**D**). PD group *N* = 71, MSA group *N* = 19, PD (−)OH group *N* = 52, PD (+)OH group *N* = 19, MSA (−)OH group *N* = 7, MSA (+)OH group *N* = 12. Whiskers represent 10th to 90th percentile. ^*^p < 0.05; ^**^p < 0.001 after Mann–Whitney *U* (**A**, **C**) or Kruskal–Wallis (**B**, **D**) testing

As depicted in Fig. 1A, patients with MSA had smaller LC volumes (mean ± SD) than PD (MSA: 2.99 mm^3^ ± 2.31; PD: 7.12 mm^3^ ± 5.87; Mann–Whitney *U* statistic 357.0, *p* = 0.002). However, SNc volumes did not significantly differ between PD and MSA (Fig. 1C; MSA: 2.99 mm^3^ ± 2.31; PD: 400.19 mm^3^ ± 168.44; Mann–Whitney *U* statistic 590.0, *p* = 0.403). Two Quade nonparametric analysis of covariances (ANCOVAs) tested for influence of, first, scanner protocol and, second, relevant clinical variables (age, disease duration, and RBDSQ group) on LC volume differences between PD and MSA. Neither test altered our findings (scanner protocol: *F* = 4.01, *p* < 0.001; age, disease duration, and RBDSQ group: *F* = 4.1, *p* = 0.046).

Comparisons of LC volumes amongst subgroups stratified by OH presence with Kruskal–Wallis testing found a significant main effect (test statistic = 16.7, *p* < 0.001). Post hoc pairwise comparisons, as shown in Fig. 1B, found that, irrespective of OH presence, MSA subgroups had smaller LC volumes than PD without OH (MSA (−)OH: 2.49 mm^3^ ± 1.54; PD (−)OH: 4.07 mm^3^ ± 3.53; test statistic = 28.7, *p* = 0.006 uncorrected, *p* = 0.038 FWE corrected; MSA ( +)OH: 3.28 mm^3^ ± 2.68; test statistic = 24.5, *p* = 0.003 uncorrected, *p* = 0.02 FWE corrected). Compared with PD (−)OH, PD (+)OH also had a smaller LC volume (PD (+)OH: 4.1 mm^3^ ± 3.5; test statistic = 18.1, *p* = 0.01 uncorrected, *p* = 058 FWE corrected). However, LC volumes for PD (+)OH and both MSA subgroups were similar (*p* > 0.3). Each MSA subgroup also had similar LC volumes (*p* = 0.736). Two Quade nonparametric ANCOVAs tested for influence of, first, scanner protocol and, second, relevant clinical variables (age, disease duration, and RBDSQ group) on LC volume differences between PD and MSA with and without OH. Neither test altered our findings (scanner protocol: *F* = 7.88.01, *p* < 0.001; age, disease duration, and RBDSQ group: *F* = 2.77, *p* = 0.047). Pairwise comparisons also found reduced LC volumes in PD (+)OH and MSA (+)OH, as compared with PD (−)OH (respectively: *t* = 2.0, *p* = 0.048; *t* = 2.0, *p* = 0.047). LC volumes in MSA (−)OH also tended to be lower compared with PD (−)OH (*p* = 0.075). Comparisons amongst PD and MSA subgroups for SNc volumes found no significant main effect (PD (−)OH: 409.68 mm^3^ ± 182.68; PD (+ OH): 374.21 mm^3^ ± 121.71; MSA (−)OH: 367.39 mm^3^ ± 79.48; MSA ( +)OH: 334.24 mm^3^ ± 122.14; Kruskal–Wallis test statistic = 1.1, *p* = 0.78), and so post hoc pairwise comparisons were not evaluated. These findings are shown in Fig. 1C and D.

A sensitivity analysis was performed to evaluate whether the single MSA-C patient included in the study biased any findings. Repeating all primary analyses with this patient removed led to the same results (Table S4, top), as did removing the four patients with PD with OH who reported use of alpha and/or beta-blocker medications (Table S4, bottom).

### Relationships between locus coeruleus volumes and orthostatic hemodynamics

**Fig. 2:**
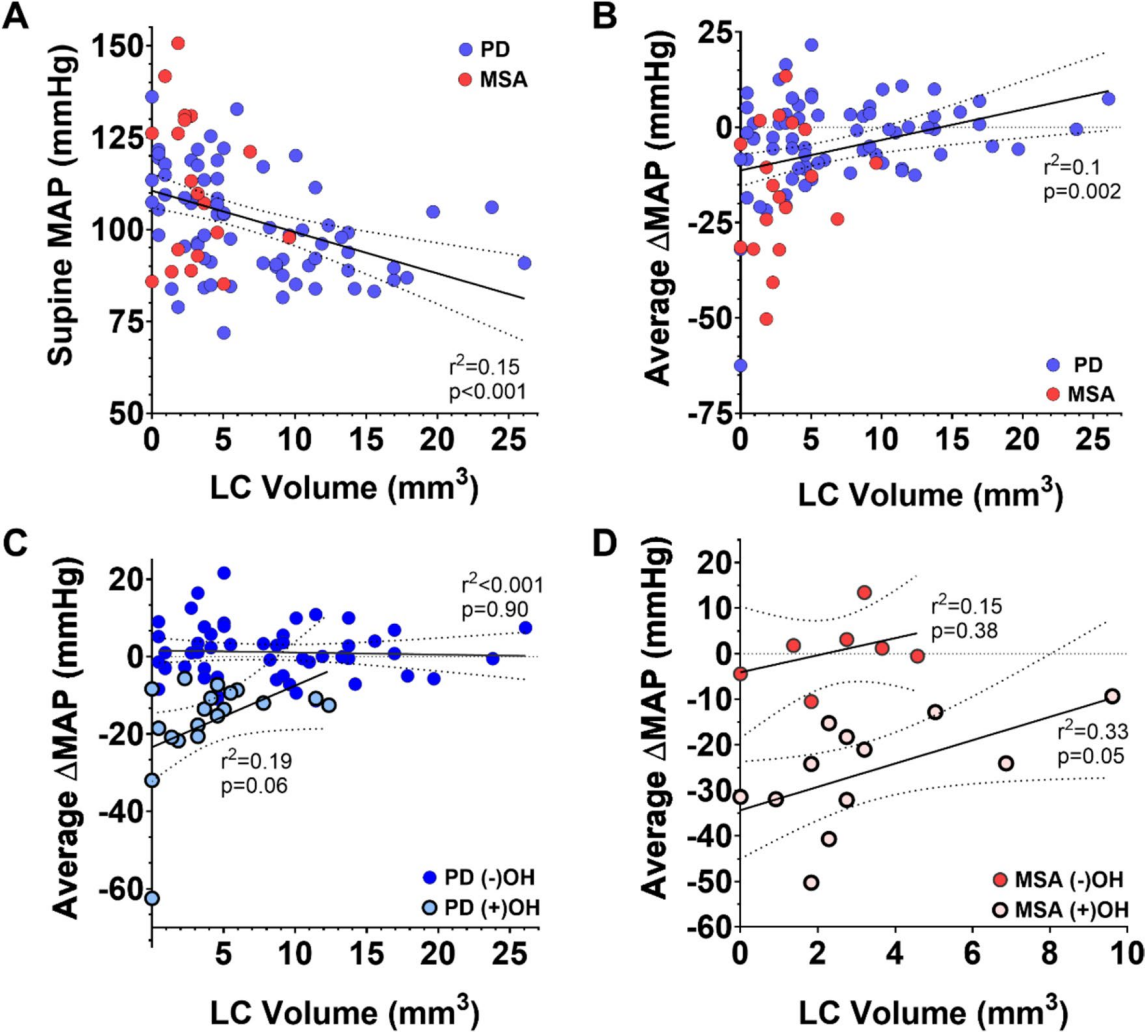
Scatter plot showing notable relationships between locus coeruleus volumes (LC, mm^3^) and supine and orthostatic change in mean arterial pressure (MAP, mmHg) across groups stratified by diagnosis (Parkinson’s disease [PD] and multiple system atrophy [MSA]) and absence (−) or presence (+) of orthostatic hypotension (OH). **A** Increased LC volume is associated with lower supine blood pressure across all subjects, driven by the PD (−)OH subgroup (see Table 2). **B** Increased LC volume is associated with orthostatic fall in MAP across all subjects. **C** Lower LC volume is associated with a greater orthostatic fall in MAP in PD (+)OH but not in PD (−)OH. **D** Lower LC volume is associated with a greater orthostatic fall in MAP in MSA (+)OH, but not in MSA (−)OH. Fit lines represent linear regression of entire sample in **A** and **B** and OH subgroups in **C** and **D**

Spearman correlations were calculated between LC volumes and (1) supine MAP and HR, and (2) average orthostatic change in MAP and change in HR across all 5 min of standing. Full results are displayed in Table 2, and notable findings are visualized in Fig. 2 as scatter plots with associated linear regression fit lines (± 95% confidence intervals) and *r*^2^ statistics. These correlations were computed across all participants, within the primary groups (PD, MSA), and within sub-groups (PD and MSA with and without OH). Across all subjects, there was a significant negative correlation between supine MAP and LC volume (rho = − 0.43, *p* < 0.001; visualized in Fig. 2A), such that larger LC volumes were associated with reduced supine MAP. This result was driven by the PD (−)OH group (rho = − 0.47, *p* < 0.001). Supine HR was positively correlated with LC volume (rho = 0.32, *p* = 0.002) across all subjects, such that greater LC volumes were associated with higher supine resting HR. This finding was also driven by the PD (−)OH group (rho = 0.32, *p* = 0.02). LC volume correlated positively across all subjects with the average orthostatic change in MAP (ΔMAP; rho = 0.31, *p* = 0.003). As visualized in Fig. 2C and D, lower LC volumes specifically in PD (+)OH (rho = 0.47, *p* = 0.04) and MSA (+)OH (rho = 0.63, *p* = 0.03) were associated with a greater orthostatic falls in MAP. There were no significant correlations between LC volume and average orthostatic HR change. Of note, these correlation patterns were maintained with sensitivity analyses whereby the one patient with MSA—cerebellar type (MSA-C) was removed (Supplementary Table S5) and when four patients with PD that reported alpha- and/or beta-blocker medication use were removed (Supplementary Table S6).

**Table 2:**
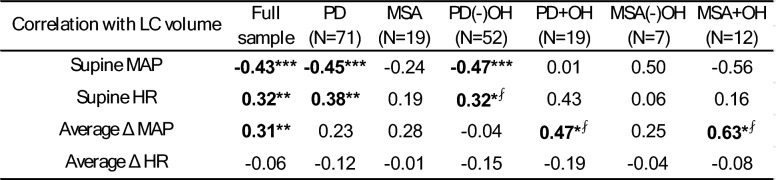
Spearman correlations between LC volume and orthostatic vitals

Δ reflects change (stand–supine), Bolded cells represent significant uncorrected two-tailed correlation (rho)

*LC* locus coeruleus, *PD* Parkinson’s disease, *MSA* multiple system atrophy, *OH* orthostatic hypotension, *MAP* mean arterial pressure, *HR* heart rate

^*^p < 0.05, ^**^p < 0.01, ^***^p < 0.001; ⨏—did not survive false discovery rate-correction threshold of 0.05

## Discussion

There is an urgent, unmet need for biomarkers that assist in the differentiation of neurodegenerative parkinsonisms. This is particularly the case for scenarios where PD presents with early OH or when MSA—parkinsonian type (MSA-P) presents without OH. Prior work has suggested that NM-MRI measurement of LC may be useful in the differential diagnosis of PD and MSA [16, 30]. However, the impact of OH status on LC volume in PD and MSA has not yet been systematically examined to our knowledge. LC projects both rostrally and caudally [56], and because its caudal noradrenergic innervation of central autonomic structures likely plays an important role in autonomic function, we hypothesized that LC neurodegeneration would be associated with OH. Thus, we predicted that LC volume would be reduced in MSA as compared with PD, because autonomic failure is more prominent in the former. Similarly, we predicted that LC neurodegeneration would be greater in patients with PD with OH, as compared with PD without OH, and that this could limit the ability of LC volume measurements to differentiate PD with OH from MSA, generally. To test whether PD with OH is associated with greater levels of localized or widespread central neurodegeneration, we also compared SNc volumes between groups, with and without OH stratification. Our predictions were generally confirmed. We additionally demonstrate that, in PD and MSA with OH, greater LC neurodegeneration correlated with greater falls in orthostatic blood pressure upon standing. These findings indicate that it is critical to consider OH status when using NM-MRI LC volume measurement as a tool to differentiate PD and MSA. In addition, these results support the notion that PD with OH is associated with a greater degree of central noradrenergic neurodegeneration than has been previously recognized.

When comparing primary groups, we found that LC volumes were significantly smaller in MSA than PD. In contrast, no difference was found between PD and MSA for SNc volumes. Prior studies, which mostly evaluated LC integrity using contrast ratio methods, rather than volume, are mixed with respect to comparisons of PD and MSA. Evaluation of LC integrity with contrast ratio methods involves selection of an ROI and determining the mean contrast within that region. A lower contrast ratio, indicative of lower NM-MRI signal from LC, represents reduced LC structural integrity. However, because LC is a small structure this approach is prone to error owing to challenges in accurately defining an ROI of LC. In this work we used an image processing and thresholding approach with established high reproducibility to measure LC and SNc volumes [54, 57]. One previous study found a greater decrease in LC contrast ratio in patients with MSA as compared with PD [23], whereas another found that MSA and PD with polysomnographically confirmed RBD both had a reduced LC contrast ratio compared with PD without RBD [24]. A third study found no difference in LC contrast ratio between PD and MSA [30], and two others reported greater reduction in LC contrast ratio in PD as compared with MSA [27, 29]. Unlike discrepancies between studies comparing LC in PD and MSA, our finding of similar SNc volumes between PD and MSA, with one exception [29], is consistent with the majority of NM-MRI studies [23, 27, 28, 30]. Aside from methodological differences (volume measurement versus contrast ratio) one possible reason for a lack of consensus amongst prior LC-based comparisons between PD and MSA is that PD phenotypes associated with a more aggressive phenotype, such as PD with OH or RBD [6, 58–60], were generally not accounted for. Specifically, studies that found no difference between PD and MSA or greater loss of LC signal in PD did not test for the impact of these phenotypes on their findings [27, 29, 30]. Indeed, when we stratified participants with PD and those with MSA into subgroups on the basis of OH presence, we found that only LC volumes for PD without OH remained larger than MSA subgroups. LC volumes for patients with PD with OH were also smaller than PD without OH. Our findings related to PD and MSA with OH were unchanged by accounting for age, disease duration, and likelihood of RBD, as well as after accounting for reported use of alpha- and/or beta-blocking medications in some patients with PD. In contrast, SNc volumes remained similar amongst all groups, irrespective of OH presence. This finding suggests that OH in PD does not predispose to a nonspecific, “diffuse malignant” increase in central neurodegeneration, per se, but instead is specifically associated with greater LC neurodegeneration [15, 35, 36]. Along with informing our understanding of the neuropathology of the PD with OH phenotype, this finding suggests the noradrenergic system is an important target for symptomatic and disease-modifying treatments and that imaging of this system may be relevant for treatment response monitoring.

Numerous studies in preclinical models implicate LC in regulation of arterial blood pressure. Stimulation of the caudal/posterior aspects of the LC directly [56] or from hemorrhage-induced volume depletion [61] is associated with activation of spinal preganglionic sympathetic vasomotor neurons and inhibition of medullary vagal nuclei [56, 62–64]. The LC is additionally activated by arterial baroreceptor unloading [65, 66] and inhibited during hypertension and stimulation of the aortic depressor nerve [67]. The rostral/anterior LC is reciprocally connected to the multiple hypothalamic nuclei [68] and stimulates neuroendocrine mechanisms that increase fluid retention and cortisol release [61, 69, 70]. Destruction of the anterior LC also leads to an increased susceptibility to vasodepressor mechanisms [61]. Further, neurotoxic loss of LC efferents leads to a reduction in diurnal plasma cortisol and an attenuated hemodynamic response to hemorrhage [70]. These findings clearly implicate the LC in arterial blood pressure regulation through direct sympathetic/pressor routes and indirectly through renal and adrenal-based mechanisms that impact volume status and vascular reactivity [71], respectively.

Greater LC neurodegeneration could contribute to cardiovascular autonomic failure in both PD and MSA by exacerbating other autonomic neurodegenerative processes. In support of this notion, we found that, in PD and MSA subgroups with OH, orthostatic falls in MAP were significantly correlated with LC volume. Two other recent studies in patients with PD demonstrated similar findings. Madelung and colleagues [35] found that reduced caudal LC contrast ratio, as measured by 7-T NM-MRI, correlated with the orthostatic fall in systolic blood pressure. Similarly, noradrenergic positron emission tomography (PET) tracer-binding uptake in the LC was negatively correlated with blood pressure falls during head-up tilt, although this finding was only observed in a mixed group of controls and patients with PD [36]. One NM-MRI study in PD, by Palermo et al. [72] and a neuropathological study by Tong and Chen [73] failed to find differences between PD with and without OH for LC-related measures. However, these two studies included fewer patients with PD with OH (Palermo, total *N* = 11; Tong and Chen, total *N* = 8) than this study and others supporting a relationship between LC integrity and orthostatic blood pressure responses. A relationship between LC integrity and OH in MSA is also consistent with prior work by Lewis and colleagues [74], who found reduced monoaminergic tracer uptake (^18^F-DOPA) in the LC of patients with MSA with OH, compared with MSA without OH. While these findings could suggest that central noradrenergic neurodegeneration and dysfunction of the LC contributes to OH in MSA, an alternative explanation could be that LC neurodegeneration progresses in parallel as MSA advances and is more likely to cause OH [75, 76]. However, because LC volumes in MSA without OH were similar to PD and MSA with OH, and because our analyses controlled for disease duration, this latter possibility seems less likely. Replicating our findings with greater numbers of MSA with/without OH, evaluating the structural integrity of other sympathetic structures in the brainstem [77], and further investigation of relationships between LC degeneration and other nonmotor manifestations of PD and MSA, such as cognition and RBD [76], would be valuable to evaluate the specificity of LC’s contribution to OH in these diseases.

Two unexpected and not previously described findings in this study were correlations between LC volume and (1) supine resting MAP and (2) supine HR. These findings further suggest that LC degeneration is associated with the emergence of cardiovascular autonomic dysfunction. Across our entire sample, there was a negative correlation between supine MAP and LC volume and a positive correlation between supine HR and LC volume. Group stratification found that these correlations were primarily driven by PD without OH. Despite prior human and nonhuman animal work showing limited impact of the LC on resting hemodynamic function, the complex neuropathological changes in PD and MSA may enhance the LC’s influence in this domain. For example, the neurodegenerative processes affecting the LC are thought to alter its inherent firing patterns, leading to high tonic activity and impaired phasic discharges in response to afferent input [78]. A more intact and tonically active LC in patients with PD without OH, who are also more likely to have intact sympathetic cardiac innervation [79], may thus promote a greater supine resting HR. This potential mechanism is consistent with preclinical optogenetic studies, which found stimulation of LC neurons increased heart rate through inhibition of the dorsal motor nucleus of the vagus and nucleus ambiguus [63]. The negative correlation between LC volume and supine MAP in the combined group of PD and MSA (with and without OH) could provide insight into the pathophysiology of neurogenic supine hypertension. This is hinted at by the marginal negative correlation in the MSA with OH group between LC volume and supine MAP (Table 2; rho = −0.56, *p* = 0.062). In both groups, a less intact and tonically active LC may be less responsive to baroreflex-mediated inhibition of its activity, raising supine blood pressure. The relationship between supine hypertension and LC integrity should be explored in future work.

### Study limitations

A few limitations to this study merit mentioning. First, formal autonomic testing was not utilized to confirm a neurogenic etiology of OH, and patients underwent orthostatic stand testing without necessarily abstaining from vasoactive medications. We attempted to account for this with strategies to increase specificity of including individuals with neurogenic OH on the basis of impairment of HR augmentation during OH or a higher BP fall threshold. In addition, our results were robust to sensitivity analyses involving censoring data of individuals who had alpha- or beta-blockers on their medication list. Nevertheless, some individuals may have had OH induced from antihypertensive medications or masked from use of anti-OH drugs. Our overall sample of PD with OH and MSA group is comparable to prior works. However, future work with larger numbers of patients with MSA without and with OH will be needed to replicate our stratified subgroup analyses. Our objectives involved comparisons of PD and parkinsonian MSA, primarily, and future work would benefit from testing whether our LC-related imaging protocol differentiates MSA-C and other neurodegenerative or genetic ataxias, particularly in the absence of early OH. Next, our patient inclusion criteria were clinically based, without neuropathological confirmation of PD and MSA diagnoses. This study included two NM-MRI protocols, which differed in some parameters. However, the differences between these protocols (number of measurements and bandwidth) had offsetting impacts on signal-to-noise ratio, mitigating this issue. Furthermore, statistical comparisons of the relative proportions of each group and subgroup scanned with each protocol were nonsignificant, and analyses including scanner protocol as a covariate did not alter our findings. Last, our scanning protocol is currently for research use alone. To make this approach broadly accessible, the corresponding author is currently pursuing US Food and Drug Administration 510 K clearance for an automated and scalable NM-MRI image processing pipeline to enable its broad use.

## Conclusions

Using NM-MRI, we found that, at a whole-group level, LC volumes, but not SNc volumes, were significantly smaller in patients with MSA, compared with PD. However, accounting for the presence of OH in PD significantly impacted the utility of LC-volume measurement to differentiate between PD and MSA. Specifically, patients with PD with OH were not significantly different from MSA with or without OH. Furthermore, lower LC volumes correlated significantly with greater falls in BP in both PD and patients with MSA with OH. These findings indicate the importance of accounting for OH when considering LC-volume measurement with NM-MRI as a potential tool to differentiate PD from MSA. Our results also suggest that central noradrenergic neurodegeneration may play a role in OH pathophysiology in both PD and MSA.

## Supplementary Information

Below is the link to the electronic supplementary material.

Supplementary file1 (DOCX 46 KB)
