# Supplementary material for: Locus coeruleus degeneration is associated with orthostatic hypotension in Parkinson’s disease and multiple system atrophy
Source: Clin Auton Res. 2025 Oct 31;36(1):53–64. doi: 10.1007/s10286-025-01164-2 (PMC12982258; doi:10.1007/s10286-025-01164-2)
Supplement: Supplementary file 1 [file 10286_2025_1164_MOESM1_ESM.docx]

Table S1 - Detailed orthostatic hemodynamic responses amongst groups

	PD (N=71)	MSA (N=19)		PD (-)OH (N=52)	PD+OH (N=19)		MSA (-)OH (N=7)	MSA+OH (N=12)	
	mean ± SD	*mean ± SD*	*p*	*mean ± SD*	*mean ± SD*	*p*	*mean ± SD*	*mean ± SD*	*p*
Supine SBP (mmHg)	140.8 ± 21.5	154.5 ± 31.8	0.13	134.6 ± 18.2	157.8 ± 21.0	**<0.001**	138.6 ± 18.9	163.8 ± 34.8	0.06
Supine DBP (mmHg)	81.9 ± 11.5	90.5 ± 16.7	**0.01**	79.4 ± 9.9	88.9 ± 12.9	**0.001**	82.1 ± 12.9	95.3 ± 17.2	0.10
Stand 1 min SBP (mmHg)	130.2 ± 21.8	129.1 ± 26.2	0.85	131.2 ± 21.0	127.5 ± 24.1	0.53	136.6 ± 22.1	124.8 ± 28.3	0.36
Stand 1 min DBP (mmHg)	80.4 ± 11.6	79.3 ± 11.9	0.82	81.1 ± 10.7	78.4 ± 13.8	0.39	83.3 ± 13.5	77.7 ± 10.9	0.10
Stand 1min MAP (mmHg)	96.8 ± 14.3	96.0 ± 15.8	0.84	97.7 ± 13.6	94.6 ± 16.4	0.43	100.9 ± 15.6	93.2 ± 15.9	0.32
Stand 1 min HR (bpm)	77.6 ± 14.2	78.0 ± 11.6	0.92	80.3 ± 13.8	70.3 ± 12.7	**0.007**	74.6 ± 8.9	80.0 ± 12.9	0.34
Stand 3 min SBP (mmHg)	131.9 ± 20.8	127.8 ± 22.7	0.46	133.5 ± 19.1	127.4 ± 25.0	0.75	138.7 ± 20.4	121.5 ± 22.4	0.14
Stand 3 min DBP (mmHg)	80.8 ± 10.7	79.9 ± 11.5	0.76	82.0 ± 9.4	77.6 ± 13.4	0.46	83.3 ± 11.7	78.0 ± 11.5	0.35
Stand 3 min MAP (mmHg)	97.7 ± 13.3	95.7 ± 14.7	0.59	99.0 ± 11.8	94.1 ± 16.6	0.75	101.6 ± 14.2	92.4 ± 14.5	0.20
Stand 3 min HR (bpm)	76.5 ± 12.9	76.9 ± 11.3	0.92	78.6 ± 12.6	70.8 ± 12.1	**0.03**	71.7 ± 8.5	79.9 ± 12.0	0.13
Stand 5 min SBP (mmHg)	131.4 ± 20.3	76.9 ± 11.3	0.44	132.7 ± 18.5	127.9 ± 24.6	0.65	139.9 ± 21.6	119.7 ± 23.8	0.08
Stand 5 min DBP (mmHg)	82.3 ± 11.8	78.4 ± 12.1	0.20	83.3 ± 10.0	79.6 ± 15.6	0.36	82.7 ± 12.9	75.8 ± 11.4	0.60
Stand 5 min MAP (mmHg)	98.5 ± 14	94.5 ± 15.5	0.27	99.6 ± 12.2	95.6 ± 18.1	0.59	101.6 ± 153.3	90.3 ± 14.6	0.10
Stand 5 min HR (bpm)	76.9 ± 12.7	76.4 ± 10.8	0.86	78.8 ± 12.4	71.7 ± 12.4	0.06	72.0 ± 5.7	78.9 ± 12.4	0.19
Avg ΔSBP 1 min (mmHg)	-10.6 ± 18.5	-25.4 ± 28.1	**0.03**	-3.3 ± 13.2	-30.3 ± 16.3	**<0.001**	-2.0 ± 10.8	-39.0 ± 26.0	**0.002**
Avg ΔDBP 1 min (mmHg)	-1.5 ± 10.5	-10.7 ± 13.4	**0.004**	1.8 ± 6.9	-10.5 ± 13.1	**<0.001**	1.1 ± 6.0	-17.7 ± 11.5	**<0.001**
Avg ΔMAP 1 min (mmHg)	-4.5 ± 12.4	-15.6 ± 16.7	**0.004**	0.1 ± 8.4	-17.1 ± 13.1	**<0.001**	0.1 ± 7.1	-24.7 ± 13.4	0.58
Avg ΔHR 1 min (mmHg)	9.3 ± 9.03	9.1 ± 6.46	0.82	10.4 ± 8.4	6.2 ± 10.1	0.08	7.1 ± 3.9	10.3 ± 7.5	0.33
Avg ΔSBP 3 min (mmHg)	-8.9 ± 17.8	-26.6 ± 27.9	**0.006**	-1.0 ± 10.5	-30.4 ± 16.0	**<0.001**	0.1 ± 10.4	-42.3 ± 22.3	**<0.001**
Avg ΔDBP 3 min (mmHg)	-1.1 ± 10.6	-10.5 ± 12.7	**0.003**	2.6 ± 6.8	-11.3 ± 12.7	**<0.001**	1.1 ± 5.6	-17.3 ± 10.5	**<0.001**
Avg ΔMAP 3 min (mmHg)	-3.7 ± 12.2	-15.8 ± 16.5	**0.003**	1.4 ± 7.0	-17.6 ± 12.6	**<0.001**	0.8 ± 7.1	-25.6 ± 11.8	**<0.001**
Avg ΔHR 3 min (mmHg)	8.2 ± 8.83	8.0 ± 6.9	0.50	8.7 ± 8.5	6.7 ± 9.9	0.41	4.3 ± 2.6	10.2 ± 7.8	0.07
Avg ΔSBP 5 min (mmHg)	-9.4 ± 18.1	-27.4 ± 32	**0.02**	-1.9 ± 12.0	-29.9 ± 15.8	**<0.001**	1.3 ± 11.1	-44.1 ± 27.9	**<0.001**
Avg ΔDBP 5 min (mmHg)	0.4 ± 10.7	-12.1 ± 13.8	**<0.001**	3.9 ± 6.3	-9.3 ± 13.9	**<0.001**	0.6 ± 7.3	-19.5 ± 10.9	**<0.001**
Avg ΔMAP 5 min (mmHg)	-2.8 ± 12.3	-17.1 ± 17.9	**<0.001**	2.0 ± 7.2	-16.1 ± 13.7	**<0.001**	0.8 ± 8.3	-27.6 ± 12.8	**<0.001**
Avg ΔHR 5 min (mmHg)	8.6 ± 8.1	7.5 ± 7.21	0.36	8.9 ± 7.5	7.6 ± 9.8	0.56	4.6 ± 4.6	9.2 ± 8.1	0.19
Avg ΔSBP (mmHg)	-9.6 ± 17.4	-26.5 ± 28.7	**0.013**	-2.1 ± 10.6	-30.2 ± 15.4	**<0.001**	-0.2 ± 10.3	-41.8 ± 24.4	**<0.001**
Avg ΔDBP (mmHg)	-0.7 ± 10.2	-11.1 ± 12.9	**0.001**	2.8 ± 6.2	-10.4 ± 12.9	**<0.001**	1.0 ± 6.1	-18.2 ± 10.3	**<0.001**

PD = Parkinson disease, MSA=multiple system atrophy, OH=orthostatic hypotension, min=minute, SBP=systolic blood pressure, DBP=diastolic blood pressure, MAP=mean arterial pressure; HR=heart rate, Δ reflects postural change subtracting stand - supine, Avg= average change in orthostatic vital across minutes 1, 3, and 5. Data are presented as mean ± standard deviation unless otherwise specified. Bold: two-tailed $p<0.05$ between groups (uncorrected). P-values shaded gray reflect independent samples T-test results. Otherwise, p-values reflect Mann-Whitney U results or Chi-square testing for categorical data.

Table S2 - Participant-reported cardiovascular medications and risk factor history

	Total study number	Patients with data available	anti-HTN Rx		anti-OH Rx		CVD		DM		HTN	
			N	%	N	%	N	%	N	%	N	%
PD	71	37	13	35.1	2	5.4	8	21.6	6	16.2	14	37.8
MSA	19	7	2	28.6	1	14.3	0	0.0	1	14.3	3	42.9
PD (-)OH	52	26	6	23.1	1	3.8	4	15.4	4	15.4	7	26.9
PD (+)OH	19	11	7	63.6	1	9.1	4	36.4	2	18.2	7	63.6
MSA (-)OH	7	3	1	33.3	0	0.0	0	0.0	0	0.0	2	66.7
MSA (+)OH	12	4	1	25.0	1	25.0	0	0.0	1	25.0	1	25.0

PD = Parkinson disease, MSA=multiple system atrophy, OH=orthostatic hypotension, Rx=medication, HTN=hypertension history, CVD=cardiovascular disease history, DM=diabetes mellitis history; 'N' reflects frequency of patients reporting the relevant variable. Percentage (%) reflects the frequency of patients with available data confirming the relevant variable.

Table S3 - Frequency distribution of NM-MRI scanning protocols

	Full sample	PD (N=71)	MSA (N=19)	PD(-)OH (N=52)	PD+OH (N=19)	MSA(-)OH (N=7)	MSA+OH (N=12)
Protocol 0	39	31	8	25	6	3	5
Protocol 1	51	40	11	27	13	4	7

Chi-Square comparisons - PD and MSA for protocol: Chi-Square value 0.02, p=0.90; groups stratified by OH presence Chi-Square value 1.56, p=0.67

**Table S4 - Sensitivity analyses**				
**Removal of MSA-C patient (MSA (+)OH)**				
Mann-Whitney Test				
			**Test Statistic**	**p**
Main Effect	PD vs MSA	LC	355.5	**0.004**
		SNc	590	0.62
Kruskal-Wallis Test				
			**Test Statistic**	**p**
Main Effect			15.5	**0.001**
Pairwise	PD (-)OH	PD (+)OH	18.0	**0.01**
		MSA (-)OH	28.6	**0.01**
		MSA (+)OH	22.0	**0.01**
	PD (+)OH	MSA (-)OH	10.6	0.35
		MSA (+)OH	4.0	0.69
	MSA (-)OH	MSA (+)OH	-6.6	0.60
**Removal of four PD (+)OH that reported beta or alpha-blocker use**				
Kruskal-Wallis Test				
			**Test Statistic**	**p**
Main Effect			15.5	**0.001**
Pairwise	PD (-)OH	PD (+)OH	14.9	**0.04**
		MSA (-)OH	27.5	**0.006**
		MSA (+)OH	23.6	**0.003**
	PD (+)OH	MSA (-)OH	12.6	0.27
		MSA (+)OH	8.77	0.36
	MSA (-)OH	MSA (+)OH	-3.86	0.75

PD=Parkinson's disease; MSA=multiple system atrophy; OH=orthostatic hypotension

**Table S5 - Spearman correlations - sensitivity analysis with MSA-C patient removed**								
Correlation		Full sample	PD (N=71)	MSA (N=18)	PD(-)OH (N=52)	PD+OH (N=19)	MSA(-)OH (N=7)	MSA+OH (N=11)
Supine MAP	LC volume	**-0.41****	**-0.45****	-0.19	**-0.47****	0.01	0.50	-0.60
Supine HR		**0.36****	**0.38****	0.39	**0.32***	0.43	0.06	0.52
Average Δ MAP		**0.29***	0.23	0.213	-0.04	**0.48***	0.25	**0.65***
Average Δ HR		-0.07	-0.12	-0.03	-0.15	-0.19	-0.04	-0.18

LC=locus coeruleus; mm3=cubic millimeter; PD=Parkinson's disease; MSA=multiple system atrophy; OH=orthostatic hypotension; MAP=mean arterial pressure; HR=heart rate; Δ=change (stand - supine); Bolded cells represent significant uncorrected two-tailed correlation (rho). *p<0.05, **p<0.01, ***p<0.001

Table S6 - Spearman correlations - sensitivity analysis with four PD (+)OH patients that reported beta and alpha-blockade use removed

Correlation		PD(-)OH (N=52)	PD+OH (N=15)	MSA(-)OH (N=7)	MSA+OH (N=12)
Supine MAP	LC volume	**-0.47****	-0.13	0.50	-0.56
Supine HR		**0.32***	0.23	0.06	0.16
Average Δ MAP		-0.04	**0.66***	0.25	**0.63***
Average Δ HR		-0.15	0.02	-0.04	-0.08

LC=locus coeruleus; mm3=cubic millimeter; PD=Parkinson's disease; MSA=multiple system atrophy; OH=orthostatic hypotension; MAP=mean arterial pressure; HR=heart rate; Δ=change (stand - supine); Bolded cells represent significant uncorrected two-tailed correlation (rho). *p<0.05, **p<0.01, ***p<0.001
